# Cross‐Sectional and Longitudinal Immunoprofiling of Oligoarticular Juvenile Idiopathic Arthritis Reveals Different Patterns in Synovial Fluid and Plasma

**DOI:** 10.1111/sji.70055

**Published:** 2025-09-26

**Authors:** Heshuang Qu, Manoj Neog, Karin Palmblad, Erik Sundberg, Alexandra Lövquist, Erik Melén, Cecilia Aulin, Helena Erlandsson Harris

**Affiliations:** ^1^ Center for Molecular Medicine, Department of Medicine Solna Karolinska Institutet Stockholm Sweden; ^2^ Division of Rheumatology Karolinska University Hospital Stockholm Sweden; ^3^ Unit of Pediatric Rheumatology Karolinska University Hospital Stockholm Sweden; ^4^ Department of Women's and Children's Health Karolinska Institutet Stockholm Sweden; ^5^ Center for Occupational and Environmental Medicine, Region Stockholm Stockholm Sweden; ^6^ Sachs Children's Hospital Stockholm Sweden; ^7^ Department of Clinical Sciences and Education, Karolinska Institutet, Södersjukhuset Stockholm Sweden; ^8^ The Broegelmann Research Laboratory, Department of Clinical Science University of Bergen Bergen Norway

**Keywords:** immunoprofiling, oligoarticular juvenile idiopathic arthritis, plasma, proteomics, synovial fluid

## Abstract

Oligoarticular juvenile idiopathic arthritis (oligoJIA) constitutes nearly 60% of all JIA cases. The immune mechanisms involved in the pathogenesis remain incompletely understood. Few proteomic studies have been performed using synovial fluid (SF) samples. We conducted an exploratory analysis of plasma and SF samples to define inflammatory profiles, assess plasma‐SF correlation and examine longitudinal variations. Using proximity extension assay (PEA), we profiled 92 immune‐related proteins in plasma and Sf from 14 oligoJIA patients (untreated or NSAID‐treated) and plasma from 28 age and sex‐matched healthy controls. Differentially expressed proteins were analysed using gene ontology (GO) and KEGG pathways via STRING. Plasma proteomic immune profiles from oligoJIA patients were highly overlapping with immune profiles of healthy donors. Six proteins were differentially expressed between the two groups. Overall, plasma and SF protein expressions correlated (*r* = 0.78), mainly driven by 13 proteins including CCL25, FGF21 and KITLG. However, the differentially expressed proteins in plasma did not correlate with those in SF. Longitudinal analysis of 20 SF and 10 plasma samples from one patient revealed immunosuppressive effects of methotrexate (MTX) with distinct kinetics in plasma and SF. Paired SF samples from five patients revealed that cell chemotaxis was a key feature in early disease, distinguishing it from the persistent phase. Immunoprofiling of SF from patients with oligoJIA identified more disease‐relevant characteristics than analysis of plasma samples. Several proteins, but not all, correlated between plasma and SF. Early‐phase enrichment of chemotaxis suggests that targeting chemokines may offer therapeutic potential for early disease remission.

## Graphical abstract

Using proximity extension assay, we determined the synovial fluid (SF) and plasma immunoprofiles of oligoJIA patients, cross‐sectionally and longitudinally. More disease‐relevant characteristics were revealed in SF than in plasma. The early‐phase enrichment of chemokines in SF suggests that targeting chemokines may offer therapeutic potential for early disease remission.

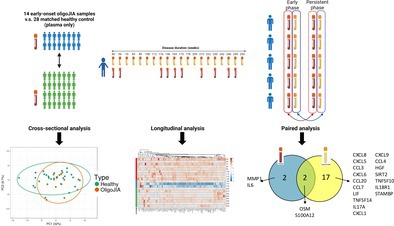

## 1 Introduction

Juvenile idiopathic arthritis (JIA) encompasses a diverse group of chronic inflammatory conditions in children, classified into seven subtypes based on clinical features, disease progression and immunological characteristics [1, 2]. A major challenge in JIA management is the absence of reliable prognostic and diagnostic biomarkers, which hampers early diagnosis and the development of personalised treatment strategies. A deeper understanding of the immunopathogenic mechanisms that are dysregulated in JIA is critical for identifying novel therapeutic targets and biomarkers. Although JIA is one of the most prevalent chronic rheumatic conditions in children, its diverse presentation often leads to misdiagnosis or underdiagnosis, highlighting the need for a better understanding of its causes.

Oligoarticular juvenile idiopathic arthritis (oligoJIA) is the most common JIA subtype, characterised by chronic inflammation involving 1 to 4 joints during the first 6 months of disease [3]. OligoJIA generally follows a benign clinical course where up to 50% display remission of disease. However, many patients continue into a chronic stage with one or a few joints affected with flares and relapses over many years [4].

While several studies have focused on individual inflammatory molecules important in pathogenesis of JIA, in particular TNF, IL1, IL6 and S100 proteins [5, 6], multiplex analyses using omics techniques remain scarce [7, 8, 9]. Early inflammatory profiling could have the potential to improve evaluation and predict outcomes in JIA [10].

Despite progress in characterisation of oligoJIA, there are still significant knowledge gaps, in particular the relationship between local and systemic inflammation and the kinetics of joint inflammation during disease progression. Such knowledge may reveal plasma biomarkers reflecting the ongoing joint inflammation, reveal new therapeutic targets and suggest optimal treatment windows for target‐specific therapies.

In this study, we investigated the inflammatory protein profile of matched plasma and SF samples from patients with oligoJIA using proximity extension assay (PEA) to quantify 92 inflammation‐related proteins. The primary aim of our study was to characterise and compare the immunoprofiles in plasma and SF. The secondary aim was to explore whether the inflammation pattern changed over time and to define immune mechanisms activated during early and late stages of oligoJIA disease course.

## 2 Materials and Methods

### 2.1 Patients

Plasma and SF samples from fourteen oligoJIA patients were collected at Astrid Lindgren's Children Hospital in Stockholm, Sweden. Paediatric rheumatologists diagnosed all patients according to the International League of Associations for Rheumatology (ILAR) criteria [3]. The patient cohort consisted of 3 males and 11 females, with ages ranging from 2 to 15 years. At the time of SF collection, all patients were either untreated or on NSAIDs only. Comprehensive clinical and laboratory data, including Global Assessment scores by Doctor (GAD) and Clinical Juvenile Arthritis Disease Activity Score of 71 joints (cJADAS‐71) were collected for each sampling occasion when available (Table 1).

**TABLE 1:** Clinical characteristics of oligoJIA patients at the sampling time points.

Patient number	Sex	Sampling age (years)	Disease duration at sampling (weeks)	Treatments at sampling	Treatment duration at sampling (weeks)	GAD (0–10)	cJADAS‐71 (0–91)
1	F	7	15	NSAIDs	6	1.0	3.0
		10	154	NSAIDs	145	1.0	2.0
2	F	12	10	NSAIDs	3	2.4	10.1
		15	150	nt	0	1.5	2.7
3	M	7	40	nt	0	1.8	5.0
		9	146	NSAIDs	65	1.8	3.0
4	F	7	17	nt	0	0.7	1.7
		9	168	NSAIDs	2	1.0	4.0
5	M	15	5	nt	0	2.1	NA
		17	154	nt	0	2.4	3.5
6	F	2	7	nt	0	1.2	NA
7	F	3	44	NSAIDs	36	1.3	7.2
8	F	7	6	NSAIDs	1	2.6	3.6
9	M	9	17	NSAIDs	7	1.8	9.0
10	F	10	14	nt	0	1.4	7.1
11	F	10	8	nt	0	1.0	2.6
12	F	13	15	nt	0	1.9	10.5
13	F	2	1	NSAIDs	1	1.7	NA
14	F	10	13	nt	0	1.1	3.7

Abbreviations: cJADAS‐71, Clinical Juvenile Arthritis Disease Activity Score of 71 joints; F, female; GAD, Global Assessment score by Doctor; M, male; NA, not available; NSAIDs, non‐steroidal anti‐inflammatory drugs; nt, no ongoing treatment.

In addition, 20 longitudinally collected SF samples and 10 longitudinally collected plasma samples from one male patient were analysed in a case study (Figure 2C).

Age‐ and sex‐matched healthy controls were obtained from a population‐based cohort (Barnens miljö‐ och hälsoundersökning [11]) collected from the same geographical region as the JIA patients and consisted of twenty‐eight plasma samples from healthy children without any chronic or inflammatory diseases.

Fresh SF and blood from both JIA patients and healthy children were collected in citrate tubes and EDTA tubes, respectively. SF samples were centrifuged at 3000 g for 10 min to obtain cell‐free samples. Blood samples were centrifuged at 3000 g for 10 to 15 min. All samples were prepared and aliquoted within 4 h and stored at −80°C until analysed.

### 2.2 Proximity Extension Assay

Undiluted plasma and SF samples were analysed using a high‐throughput, multiplex immunoassay (Proseek Multiplex, Proximity Extension Assay (PEA) technology, Inflammation panel, Olink Bioscience, Sweden). The selected panel includes 92 immune‐related proteins, primarily cytokines and chemokines, as listed in Table S1. The assay utilises epitope‐specific binding and hybridization of a set of paired oligonucleotide antibody probes, which are subsequently amplified using quantitative PCR, resulting in log base‐2 normalised protein expression (NPX) values. Proteins with a call rate below 20% were excluded from further analysis, resulting in 69 proteins in plasma and 73 proteins in SF being included.

### 2.3 Data Processing and Statistical Analysis

In the cross‐sectional analysis of plasma samples from healthy controls and oligoJIA, ordinary two‐way ANOVA was performed on oligoJIA (*n* = 14) and age‐ and sex‐healthy controls (*n* = 28). Pearson correlation analysis was performed on matched plasma (*n* = 11) and SF (*n* = 11) samples, isolated from individual patients at the same clinical visit times. For paired analysis of plasma and SF samples from the early and late disease stages, ordinary two‐way ANOVA was performed on the early (*n* = 5) and late stage (*n* = 5). All statistical analyses were corrected for multiple comparisons by controlling the False Discovery Rate (FDR) via the two‐stage step‐up method of Benjamini, Krieger and Yekutieli. Adjusted *p*‐values less than 0.05 were regarded as significant. The tests were performed by GraphPad Prism version 9.4.1 (San Diego, CA, USA).

Hierarchical Clustering Analysis (HCA) and Principal Component Analysis (PCA) were performed by ClustVis Web Tool [12] to visualise the clustering of samples. For the HCA map of the cross‐sectional analysis, unit variance scaling was applied to rows; rows were clustered using correlation distance and average linkage; columns were clustered using Euclidean distance and average linkage. For the HCA map of the case study analysis, unit variance scaling was applied to rows; columns were clustered using correlation distance and average linkage. Protein–protein interaction networks, GO process analysis and KEGG pathway analysis were performed by STRING 11.5 [13]. Sankey diagrams were generated using the pySankey package in Python 3.

## 3 Results

Summaries of demographic and disease characteristics of the patients and age and sex distribution of patients and healthy controls are outlined in Tables 1 and 2.

**TABLE 2:** Average age of patients and healthy controls.

	Average age^b^ (years ± SD)	Average age of females^b^ (years ± SD)	Average age of males^b^ (years ± SD)
Patients (*n* = 14; F = 11, M = 3)	8.14 ± 3.96	7.55 ± 3.88	10.33 ± 4.16
Healthy controls (*n* = 28; F = 22, M = 6)	8.00 ± 2.67	7.64 ± 2.74	9.33 ± 2.07
*p* value^a^	0.904	0.945	0.725

^a^ Statistics: Unpaired *t*‐test with Welch's correction.

^b^ Average age at the first sample occasion. Five patients had samples obtained both in the early phase and in the persistent phase; data included in the table are from the early phase sampling.

### 3.1 Overlapping Proteomic Immunoprofiles in Plasma From oligoJIA and Age and Sex‐Matched Healthy Controls

**FIGURE 1:**
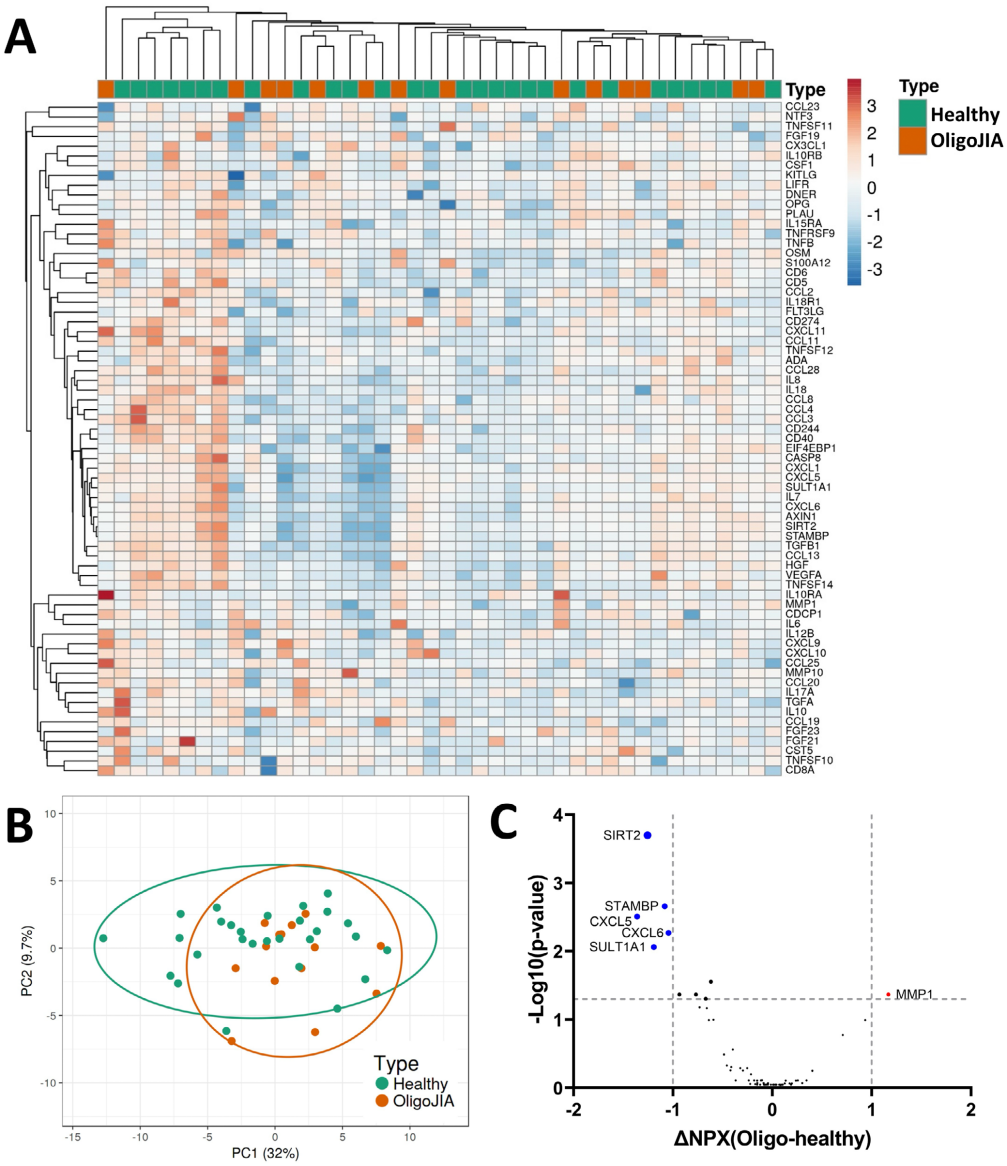
Cross‐sectional analysis of plasma immunoprofiles in patients with OligoJIA and in matched healthy controls revealed overlapping profiles. (A) HCA plot of 14 patients and 28 age‐ and sex‐matched healthy controls. Unit variance scaling was applied to rows; Rows are clustered using correlation distance and average linkage; Columns were clustered using Euclidean distance and average linkage. (B) PCA of the two groups based on the 69 proteins shown in (A) displayed a major overlap between oligoJIA and healthy. The confidence level of the ellipses is 0.95. (C) Volcano plot of differentially expressed proteins comparing oligoJIA and healthy controls. X‐axis represent the subtraction between oligoJIA and healthy control. One unit difference in NPX value represents a doubling of the actual concentration. Blue dots represent proteins which are significantly lower in plasma from patients with oligoJIA than healthy controls (*p* < 0.05 and ΔNPX<‐1.0). Red dots represent proteins which are significantly higher in plasma from patients with oligoJIA than healthy controls (*p* < 0.05 and ΔNPX< −1.0 or > 1.0). All statistical analyses were corrected for multiple comparisons by controlling the False Discovery Rate (FDR) via the two‐stage step‐up method of Benjamini, Krieger and Yekutieli.

We first investigated the inflammation‐related protein profiles in plasma from patients with oligoJIA by comparing them with age‐ and sex‐matched healthy controls. The patients were treatment naïve, that is, no ongoing medication or only NSAIDs (Table 1). HCA and PCA plots revealed a major overlap in inflammatory profiles between oligoJIA and healthy controls based on the 69 measured proteins (Figure 1A,B). Five proteins, SIRT2, STAMBP, CXCL5, CXCL6 and SULT1A1, were significantly lower in oligoJIA than in healthy controls (Figure 1C). One protein, MMP1, was significantly higher in oligoJIA than in healthy controls (Figure 1C). Full data sets are presented in Table S2.

### 3.2 Immunoprofiles in Plasma and SF From oligoJIA Displayed Overall Positive Correlation

**FIGURE 2:**
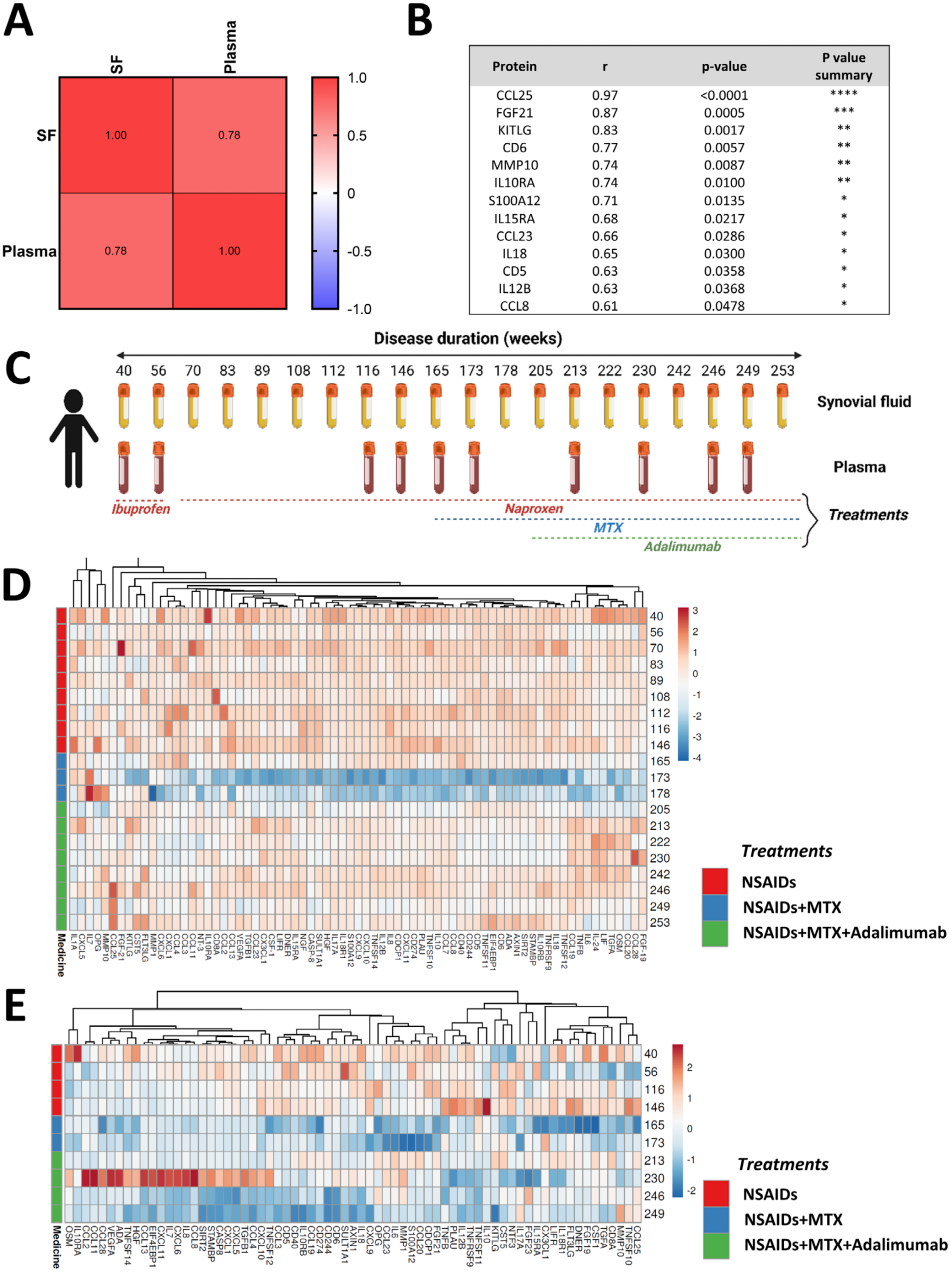
Immunoprofiles in plasma and SF from oligoJIA patients. (A) Pearson correlation analysis indicated that plasma and SF from patients (*n* = 14) who were treatment naïve or treated with NSAIDs only were positively correlated, with an *R* value of 0.78. (B) 13 out of 69 proteins were significantly correlated between plasma and SF. (C) Illustration of the sampling times and treatments of the single patient case study. (D) HCA plot of the single patient case study longitudinal 20 SF samples analysed by PEA. (E) HCA plot of the single patient case study longitudinal 10 plasma samples analysed by PEA. In both (D) and (E) the unit variance scaling was applied to rows; columns were clustered using correlation distance and average linkage.

Patients with oligoJIA have manifestations in one or several joints and usually do not display quantifiable systemic inflammation. While plasma sampling is commonly used for clinical diagnosis, SF is not. Therefore, to investigate if the systemic protein composition of plasma reflects the local protein composition in SF, we compared paired plasma and SF samples isolated at the same sampling occasion from 11 patients. Multiple correlation analysis indicated that plasma and SF displayed an overall positive correlation, with an *R* value of 0.78 (Figure 2A). This was primarily influenced by 13 proteins, and all significant correlations were positive (Figure 2B). However, none of the differentially expressed plasma proteins comparing oligoJIA and healthy controls correlated between plasma and SF in oligoJIA patients (Table S2).

### 3.3 Immunoprofiles in Longitudinal SF and Plasma Samples From One Patient

To investigate if the inflammatory pattern fluctuates over time in oligoJIA, we analysed the immunoprofile of one patient using 20 SF samples and 10 plasma samples collected over a 4‐year time period. The patient was treated with NSAIDs over the course of the sampling, initially ibuprofen, but switched to naproxen at week 97. From week 165, treatment with MTX was added, and at week 205, TNF inhibiting treatment was initiated. Additionally, local cortisone injections were given; these were done immediately following SF withdrawal. Due to recurrent joint inflammation, cortisone injections and SF withdrawals were done on numerous occasions (Figure 2C).

Immune profiles of the 20 SF samples (Figure 2D) and 10 plasma samples (Figure 2E) revealed different clustering of investigated proteins in SF as compared to blood. MTX treatment resulted in reduced levels of most proteins included in the panel. These effects were detected earlier in plasma (week 165) than in SF (week 173). Neither NSAIDs nor TNF inhibitor led to shifts in the SF immunoprofiles as strong as MTX. The pattern in plasma was more varied, indicating the correlation between plasma and SF is influenced by medication.

The clinical parameters, including C‐reactive protein (CRP), erythrocyte sedimentation rate (ESR), GAD and cJADAS‐71, were recorded at the times of sampling and are listed in Figure S1. CRP and ESR were elevated compared to clinical cut‐offs (Figure S1A,B). GAD and JADAS scores showed that, though the patient had mild inflammation with respect to the clinical parameters, the scores peaked independent of the anti‐TNF treatment (Figure S1C,D).

### 3.4 Analysis of Early Stage oligoJIA SF Samples Showed Enrichment in Immune Cell Chemotaxis

As the longitudinal data from the single case study patient indicated that the inflammatory protein profile was stable during NSAID treatment, we sought to further explore whether there are any differences between early and persistent disease phases. Hence, a cross‐sectional analysis of five treatment‐naive patients (no treatment or NSAID) was performed. Paired plasma and SF samples during early (disease duration 17.4 ± 13.5 weeks) and late disease stages (disease duration 154.4 ± 8.3 weeks) were analysed with PEA.

**FIGURE 3:**
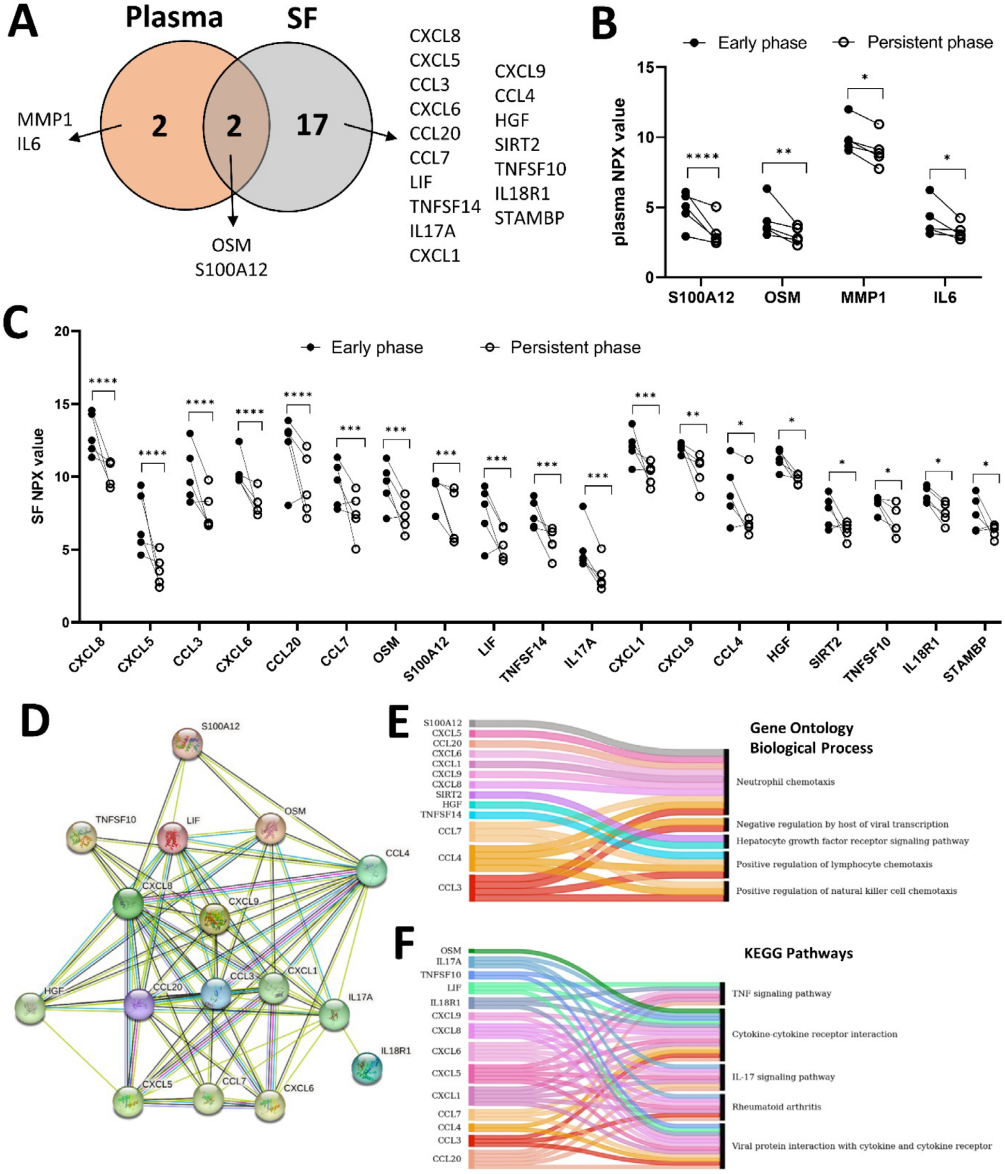
Paired analysis of early and late stages of oligoJIA showed enrichment in immune cell chemotaxis in SF at the early stage. Comparison of plasma and SF protein levels in paired samples from five patients during early (disease duration 17.4 ± 13.5 weeks) and late disease stages (disease duration 154.4 ± 8.3 weeks). All patients were treatment naïve or treated with NSAIDs only. (A) The Venn diagram shows the overlap of significantly differently expressed proteins between early and late stages in plasma and in SF. (B) Individual, paired NPX‐values for the 4 significantly increased plasma proteins during the early stage. (C) Individual, paired NPX‐values for the 19 significantly increased proteins in SF during early and late disease stages. (D) Protein network generated by STRING based on the 19 significantly different proteins in SF. (E) GO analysis and (F) KEGG pathway analysis based on the 19 significantly different proteins in SF. Statistical analysis (B, C) was performed by ordinary two‐way ANOVA.

The results showed that 4 and 19 proteins were differentially expressed comparing the early and late disease stages in plasma and SF, respectively. Two proteins, OSM and S100A12, differed in both SF and plasma (Figure 3A). All the differently expressed proteins were lower in the late stage than in the early stage (Figure 3B,C). Based on the 19 differentially expressed proteins in SF, we generated a protein network using STRING (Figure 3D). This indicated that the major, central protein hubs mainly were chemokines, such as CXCLs and CCLs. Similarly, gene ontology (GO) analysis showed that the top five enriched biological pathways were ‘Positive regulation of natural killer cell chemotaxis’, ‘Positive regulation of lymphocyte chemotaxis’, ‘Hepatocyte growth factor receptor signalling pathway’, ‘Negative regulation by host of viral transcription’ and ‘Neutrophil chemotaxis’ (Figure 3E). KEGG analysis showed that the top five enriched pathways were ‘Viral protein interaction with cytokine and cytokine receptor’, ‘Rheumatoid arthritis’, ‘IL‐17 signalling pathway’, ‘Cytokine‐cytokine receptor interaction’ and ‘TNF signalling pathway’ (Figure 3F). Detailed pathway strength and *p* values corrected for multiple testing within each category using the Benjamini–Hochberg procedure are listed in Tables S3 and S4.

## 4 Discussion

In this exploratory study, we set out to define inflammatory mediators and pathways involved in the pathogenesis of oligoJIA using a proteomics approach. We took advantage of our collection of paired SF and plasma samples collected in parallel for over 200 weeks. Additionally, we used paired SF and plasma samples from early and late stages of oligoJIA to explore whether inflammatory profiles differed as the disease progressed.

Compared with age‐ and sex‐matched healthy controls, the inflammation‐related plasma proteome in patients with oligoJIA showed only minor differences at the individual protein level. This result reflects the clinical picture, as patients with oligoJIA primarily display local joint inflammation with limited systemic symptoms. We found that 6 out of 69 proteins (MMP1, SIRT2, STAMBP, CXCL5, CXCL6 and SULT1A1) were significantly differentially expressed in oligoJIA compared to healthy control plasma.

Elevated levels of the collagenase MMP1 have been observed in SF from patients with rheumatoid arthritis (RA) [14]. However, few studies have analysed MMP1 levels in JIA plasma. Wojdas et al. found significantly higher levels of MMP1 in plasma in JIA patients than in healthy controls [15]. Similarly, we found significantly higher levels of MMP1 in plasma in patients with active systemic JIA (sJIA) than in healthy controls [16]. As multiple cell types produce MMPs, the origin of the recorded MMP1 could be both from the inflamed joints and peripheral tissues.

SIRT2 is a cytosolic NAD+ (nicotinamide adenine dinucleotide)‐dependent deacetylase. In collagen‐induced arthritis, a deficiency of *SIRT2* resulted in a more severe arthritic phenotype [16]. Secretion of SIRT2 from activated myeloid cells in tumour microenvironments has been reported and associated with the promotion of metastasis formation through the deacetylation of extracellular proteins [17]. Increased serum levels are reported for lung cancer patients [18]. We found SIRT2 to be lower in oligoJIA patients than in healthy controls, potentially indicating the importance of SIRT2 in oligoJIA pathogenesis.

STAMBP is an endosome‐associated deubiquitinase. It has been implicated in the modulation of NLRP3 cytokine secretion [19] but not investigated for a pathogenic role in arthritis. SULT1A1 is a cytosolic sulfotransferase with known impact on drug metabolism but unexplored in arthritis.

CXCL5 and CXCL6 are chemokines and ligands of CXCR2. It has been demonstrated that fibroblast‐like synoviocytes secrete CXCL5 upon activation, leading to neutrophil recruitment to the joints [20]. Koch et al. found elevated CXCL5 levels in both SF and plasma from RA compared with other forms of arthritis and healthy controls [21]. In contrast, decreased levels of serum CXCL5 have been reported in SLE and were negatively correlated with disease activity [22].

In a previous study, we observed that plasma CXCL5 levels were dynamically regulated, increasing during active disease and decreasing during inactive phases as compared to healthy controls [8]. Together with the finding of lower plasma levels of CXCL5 and CXCL6 in oligoJIA, our present studythis suggests their involvement in underlying inflammatory pathways and potential disease‐associated fluctuation. This warrants further investigation and validation.

To investigate the inflammatory process over time, a case study of a patient with persistent oligoJIA with 20 sampling occasions over a 4‐year period and a sub‐cohort of longitudinal samples from 5 patients was analysed. The case study patient was diagnosed with oligoJIA having knee joint inflammation. Systemic medication with NSAIDs was not efficient in treating the disease, as joint swelling was not prevented. The disease was considered mild since it only involved one knee joint, and intra‐articular treatment with cortisone caused remission for varying lengths of time. Considering the potential side effects of MTX and anti‐TNF treatment, the patient had NSAIDs only treatment until week 146 after disease onset when MTX was prescribed. As resistance to MTX treatment developed, a switch to TNF‐blockade was prescribed. The washout period for intra‐articular cortisone is considered to be 12 weeks. Most sample occasions were more than 12 weeks apart, although even at the sampling occasions closer than 12 weeks, no major differences were recorded by HCA. Although this patient might not be considered a typical oligoJIA patient, recurrent joint inflammation occurs in a number of oligoJIA patients and by immunoprofiling a longitudinal series of plasma and SF from one individual, we could map the joint inflammation at the molecular level. We observed a persistent inflammatory protein immunoprofile in SF, especially during the NSAIDs treatment period. Interestingly, though MTX was not effective from a clinical perspective as joint swelling reappeared, it did result in a general reduction of inflammatory proteins in both plasma and SF. The effects of MTX were however transient, as downregulation of protein levels started to reverse after 5 weeks. This could possibly be due to the development of MTX resistance, which occurs in up to 65% of patients with JIA [23]. Despite multiple studies investigating the mechanisms behind MTX resistance, no specific predictive marker of MTX efficacy or adverse events has yet been identified. Our case study and the transient effect on the protein profiles suggest that immunoprofiling, especially of SF, may be useful for clinicians to adjust medicine prescription in time to improve disease more efficiently. An evaluation of a larger cohort would be required to validate the utility of our measurements.

Despite a positive correlation between plasma and SF measurements being observed in the analysis of the 11 treatment‐naïve oligoJIA, in the case study, the immunoprofile in SF was not reflected in the plasma. For example, at week 165, when we had not mapped any major changes in the SF profile, we could already record an evident inflammation reduction in the plasma profile. Additionally, after adding anti‐TNF treatment, there was no shift in the SF profile, while some up‐ and downregulation could be detected in plasma. In conclusion, at the individual level, the local inflammation profile recorded in SF could not be reproduced in plasma samples. It further implies the necessity to study SF samples to reveal dysregulated immune mechanisms contributing to the local inflammation in joints.

Although the case study described a non‐fluctuating immunoprofile over time, the paired analysis of the early and late stages in five patients revealed changes in the immune profile with disease progression. Specifically, a decrease in chemokines and cytokines was observed over time, supported by the enriched pathways of cell chemotaxis from GO and KEGG analysis. STRING analysis of the DEPs placed CXCL and CCL chemokines as central hubs in the immune network. Our data support the current view that the initiation of the JIA pathophysiological cascade includes abnormal infiltration and activation of immune cells in the joints, where the cells produce pro‐inflammatory mediators that cause joint destruction and systemic complications [24]. We then observed a decrease in all markers in the late stage. Therefore, we speculate that with oligoJIA progression, the immune cell infiltration becomes less predominant; instead, local joint cells, such as synovial fibroblasts and macrophages, assume the disease‐driving role leading to further joint inflammation and joint destruction.

Numerous chemokines and their receptors have been detected in arthritic synovium [25, 26]; various strategies, including peptides and other small molecule inhibitors of chemokines and their receptors, have given promising results in preclinical studies [27, 28] but recent clinical trials using antibodies and synthetic compounds have failed. Several aspects might interfere with the efficacy of chemokine and chemokine receptor blockade, including the redundancy of the chemokine system, the interference with homeostatic function, and the administration time point during the disease course. Despite the discouraging clinical trial results, more studies could be performed to investigate the impact of treatment administration time and frequency. Based on our findings, it is possible that targeting chemokines during the early disease phase would be more effective in inhibiting disease progression than after long‐term disease.

The limitations of this study include the small patient cohort and lack of comparison with SF from non‐arthritic controls. Collection of SF from healthy individuals and, in particular, children is rare as it is considered not ethically justified. Further, we used a targeted PEA panel measuring 92 inflammatory‐related proteins, which is a small proportion of all the proteins in plasma and SF. Some mechanisms, for example, MTX resistance, were not explained by the current results. In future studies, verification of our findings in a larger cohort and by techniques covering more of or even the entire proteome is highly warranted.

In conclusion, by applying a high‐throughput and multiplex immunoassay, we have defined plasma and SF inflammatory protein profiles in an oligoJIA cohort. We found that patients with oligoJIA showed similar plasma immunoprofiles as age and sex‐matched healthy controls. We performed a longitudinal analysis of a series of plasma and SF samples from an individual oligoJIA case identifying a persistent inflammatory profile over time. A decrease in inflammation was observed by MTX treatment but was not reflected in the clinical symptoms. When comparing early and late disease stages of oligoJIA in a subgroup of our cohort, we found that chemokines and chemotaxis were important contributors early in the disease course and then reduced with the disease progression. Our data indicate chemokines as potential oligoJIA therapeutic targets, especially during the early disease phase. Furthermore, our study underlines the importance of SF samples, or synovial tissue samples, when studying immunopathogenic mechanisms active during oligoJIA.

## Author Contributions

H.E.H., H.Q., E.S., C.A. and M.N. conceived the study. H.E.H., E.S. and K.P. recruited the patients. E.S. and K.P. collected patient samples and recorded the clinical scores. H.Q., K.P., E.S., C.A., M.N. and H.E.H. analysed the data. H.Q. and M.N. wrote the first draft of the manuscript. H.Q., H.E.H. and C.A. produced the illustrations and wrote the final draft of the manuscript. All authors critically reviewed and approved the final version of the manuscript.

## Ethics Statement

The study was performed in accordance with the Declaration of Helsinki and ethical approvals were obtained from the North Ethical Committee in Stockholm, Sweden (Dnrs 2009–1139‐30‐4 and 2010–165–31‐2 for Juvenile Arthritis BioBank Astrid Lindgren's hospital and Dnr 03–067 for Barnens miljö‐och hälsoundersökning).

## Consent

Informed consent was given by all the sample donators and their parents to participate in the study and for dissemination of the results.

## Conflicts of Interest

The authors declare no conflicts of interest.

## Supporting information


**Table S1:** List of all 92 biomarkers analysed in Olink inflammatory panel. Call rate represents the proportion of samples with measurable concentrations above the limit of detection. Proteins with a low call rate below 20% were excluded from further the analysis.
**Table S2:** Detailed fold change and *p* values of oligoJIA‐healthy control cross‐sectional analysis.
**Table S3:** Gene ontology (GO) analysis results based on the 19 significantly different SF proteins between early and persistent phase of oligoJIA.
**Table S4:** Kyoto Encyclopedia of Genes and Genomes (KEGG) analysis results based on the 19 significantly different SF proteins between early and persistent phase of oligoJIA.
**Figure S1:** Clinical parameters of the case study. The clinical measurements, including C‐reactive protein (CRP) (A), erythrocyte sedimentation rate (ESR) (B), Global Assessment scores by Doctor (GAD) (C) and Clinical Juvenile Arthritis Disease Activity Score of 71 joints (cJADAS‐71) (D), were listed. Based on the age of the patient, the limitation and range of each parameter are: CRP higher than 10 mg/L or ESR higher than 13 mm/h is regarded as inflammation. GAD score ranges between 0 and 10. cJADAS‐71 score ranges between 0 and 91, the high, low and inactive disease activity cut‐off are 10.5, 3.8 and 1.0, separately.

## Data Availability

The data that supports the findings of this study are available in the Supporting Information of this article.
