# Supplementary material for: Cross‐Sectional and Longitudinal Immunoprofiling of Oligoarticular Juvenile Idiopathic Arthritis Reveals Different Patterns in Synovial Fluid and Plasma
Source: Scand J Immunol. 2025 Sep 26;102(4):e70055. doi: 10.1111/sji.70055 (PMC12475091; doi:10.1111/sji.70055)
Supplement: Supplementary file 1 — Table S1: List of all 92 biomarkers analysed in Olink inflammatory panel. Call rate represents the proportion of samples with measurable concentrations above the limit of detection. Proteins with a low call rate below 20% were excluded from further the analysis. Table S2: Detailed fold change and p values of oligoJIA‐healthy control cross‐sectional analysis. Table S3: Gene ontology (GO) analysis results based on the 19 significantly different SF proteins between early and persistent phase of oligoJIA. Table S4: Kyoto Encyclopedia of Genes and Genomes (KEGG) analysis results based on the 19 significantly different SF proteins between early and persistent phase of oligoJIA. Figure S1: Clinical parameters of the case study. The clinical measurements, including C‐reactive protein (CRP) (A), erythrocyte sedimentation rate (ESR) (B), Global Assessment scores by Doctor (GAD) (C) and Clinical Juvenile Arthritis Disease Activity Score of 71 joints (cJADAS‐71) (D), were listed. Based on the age of the patient, the limitation and range of each parameter are: CRP higher than 10 mg/L or ESR higher than 13 mm/h is regarded as inflammation. GAD score ranges between 0 and 10. cJADAS‐71 score ranges between 0 and 91, the high, low and inactive disease activity cut‐off are 10.5, 3.8 and 1.0, separately. [file SJI-102-e70055-s001.docx]

##

## **Supplementary Table S1. List of all 92 biomarkers analyzed in Olink inflammatory panel.**

Call rate represents the proportion of samples with measurable concentrations above the limit of detection. Proteins with a low call rate below 20% were excluded from further the analysis

| **ID**  **(Olink ID if different)** | **Entrez Gene Name** | **Type(s)** | **Call rate in plasma**  **(%)** | **Call rate in synovial fluid (%)** |
| --- | --- | --- | --- | --- |
| ADA | adenosine deaminase | enzyme | >80% | >80% |
| ARTN | artemin | growth factor | <20% | <20% |
| AXIN1 | axin 1 | other | >80% | >80% |
| CASP8 | caspase 8 | peptidase | 20-80% | >80% |
| CCL11 | C-C motif chemokine ligand 11 | cytokine | >80% | >80% |
| CCL13 (MCP-4) | C-C motif chemokine ligand 13 | cytokine | >80% | >80% |
| CCL19 | C-C motif chemokine ligand 19 | cytokine | >80% | >80% |
| CCL2 (MCP-1) | C-C motif chemokine ligand 2 | cytokine | >80% | >80% |
| CCL20 | C-C motif chemokine ligand 20 | cytokine | >80% | >80% |
| CCL23 | C-C motif chemokine ligand 23 | cytokine | >80% | >80% |
| CCL25 | C-C motif chemokine ligand 25 | cytokine | >80% | >80% |
| CCL28 | C-C motif chemokine ligand 28 | cytokine | 20-80% | >80% |
| CCL3 | C-C motif chemokine ligand 3 | cytokine | >80% | >80% |
| CCL4 | C-C motif chemokine ligand 4 | cytokine | >80% | >80% |
| CCL7 (MCP-3) | C-C motif chemokine ligand 7 | cytokine | <20% | >80% |
| CCL8 (MCP-2) | C-C motif chemokine ligand 8 | cytokine | >80% | >80% |
| CD244 | CD244 molecule | transmembrane receptor | >80% | >80% |
| CD274 (PD-L1) | CD274 molecule | enzyme | >80% | >80% |
| CD40 | CD40 molecule | transmembrane receptor | >80% | >80% |
| CD5 | CD5 molecule | transmembrane receptor | >80% | >80% |
| CD6 | CD6 molecule | transmembrane receptor | >80% | >80% |
| CD8A | CD8a molecule | other | >80% | >80% |
| CDCP1 | CUB domain containing protein 1 | other | >80% | >80% |
| KITLG (SCF) | c-Kit ligand | growth factor | >80% | >80% |
| CSF1 | colony stimulating factor 1 | cytokine | >80% | >80% |
| CST5 | cystatin D | other | >80% | >80% |
| CX3CL1 | C-X3-C motif chemokine ligand 1 | cytokine | >80% | >80% |
| CXCL1 | C-X-C motif chemokine ligand 1 | cytokine | >80% | >80% |
| CXCL10 | C-X-C motif chemokine ligand 10 | cytokine | >80% | >80% |
| CXCL11 | C-X-C motif chemokine ligand 11 | cytokine | >80% | >80% |
| CXCL5 | C-X-C motif chemokine ligand 5 | cytokine | >80% | >80% |
| CXCL6 | C-X-C motif chemokine ligand 6 | cytokine | >80% | >80% |
| CXCL9 | C-X-C motif chemokine ligand 9 | cytokine | >80% | >80% |
| DNER | delta/notch like EGF repeat containing | transmembrane receptor | >80% | >80% |
| EIF4EBP1 (4E-BP1) | eukaryotic translation initiation factor 4E binding protein 1 | translation regulator | >80% | >80% |
| FGF19 | fibroblast growth factor 19 | growth factor | >80% | >80% |
| FGF21 | fibroblast growth factor 21 | growth factor | >80% | 20-80% |
| FGF23 | fibroblast growth factor 23 | growth factor | 20-80% | <20% |
| FGF5 | fibroblast growth factor 5 | growth factor | <20% | <20% |
| FLT3LG (Flt3L) | fms related tyrosine kinase 3 ligand | cytokine | >80% | >80% |
| GDNF | glial cell derived neurotrophic factor | growth factor | <20% | <20% |
| HGF | hepatocyte growth factor | growth factor | >80% | >80% |
| IL10 | interleukin 10 | cytokine | 20-80% | >80% |
| IL10RA | interleukin 10 receptor subunit alpha | transmembrane receptor | 20-80% | 20-80% |
| IL10RB | interleukin 10 receptor subunit beta | transmembrane receptor | >80% | >80% |
| IL12B | interleukin 12B | cytokine | >80% | >80% |
| IL13 | interleukin 13 | cytokine | <20% | <20% |
| IL15RA | interleukin 15 receptor subunit alpha | transmembrane receptor | >80% | 20-80% |
| IL17A | interleukin 17A | cytokine | 20-80% | >80% |
| IL17C | interleukin 17C | cytokine | <20% | <20% |
| IL18 | interleukin 18 | cytokine | >80% | >80% |
| IL18R1 | interleukin 18 receptor 1 | transmembrane receptor | >80% | >80% |
| IL1A | interleukin 1 alpha | Extracellular Space | <20% | 20-80% |
| IL2 | interleukin 2 | Extracellular Space | <20% | <20% |
| IL20 | interleukin 20 | Extracellular Space | <20% | <20% |
| IL20RA | interleukin 20 receptor subunit alpha | Plasma Membrane | <20% | <20% |
| IL22RA1 | interleukin 22 receptor subunit alpha 1 | Plasma Membrane | <20% | <20% |
| IL24 | interleukin 24 | Extracellular Space | <20% | 20-80% |
| IL2RB | interleukin 2 receptor subunit beta | Plasma Membrane | <20% | <20% |
| IL33 | interleukin 33 | Extracellular Space | <20% | <20% |
| IL4 | interleukin 4 | Extracellular Space | <20% | <20% |
| IL5 | interleukin 5 | Extracellular Space | <20% | <20% |
| IL6 | interleukin 6 | Extracellular Space | 20-80% | >80% |
| IL7 | interleukin 7 | Extracellular Space | 20-80% | 20-80% |
| IL8 | C-X-C motif chemokine ligand 8 | Extracellular Space | >80% | >80% |
| INFγ | Interferon gamma | Extracellular Space | NA* | NA* |
| LIF | LIF interleukin 6 family cytokine | Extracellular Space | <20% | >80% |
| LIFR | LIF receptor subunit alpha | Plasma Membrane | >80% | >80% |
| MMP1 | matrix metallopeptidase 1 | Extracellular Space | >80% | >80% |
| MMP10 | matrix metallopeptidase 10 | Extracellular Space | >80% | >80% |
| NGF (Beta-NGF) | nerve growth factor | Extracellular Space | <20% | 20-80% |
| NRTN | neurturin | Extracellular Space | <20% | <20% |
| OPG | TNF receptor superfamily member 11b | Plasma Membrane | >80% | 20-80% |
| OSM | oncostatin M | Extracellular Space | >80% | >80% |
| PLAU (uPA) | Urolinase-type plasminoen activator | Extracellular Space | >80% | >80% |
| S100A12 (EN-RAGE) | S100 calcium binding protein A12 | Cytoplasm | >80% | >80% |
| SIRT2 | sirtuin 2 | Nucleus | 20-80% | >80% |
| SLAMF1 | signaling lymphocytic activation molecule family member 1 | Plasma Membrane | <20% | >80% |
| NTF3 (NT-3) | Neurotrophin 3 | Extracellular Space | 20-80% | <20% |
| STAMBP | STAM binding protein | Nucleus | >80% | >80% |
| SULT1A1 (ST1A1) | sulfotransferase family 1A member 1 | Cytoplasm | 20-80% | >80% |
| TGFA | transforming growth factor alpha | Extracellular Space | >80% | >80% |
| TGFB1 (LAP TGF-beta-1) | transforming growth factor beta 1 | Extracellular Space | >80% | >80% |
| TNF | Tumor necrosis factor | Extracellular Space | NA* | NA* |
| TNFB | lymphotoxin alpha | Extracellular Space | >80% | >80% |
| TNFRSF9 | TNF receptor superfamily member 9 | Plasma Membrane | >80% | >80% |
| TNFSF10 (TRAIL) | TNF superfamily member 10 | Extracellular Space | >80% | >80% |
| TNFSF11 (TRANCE) | TNF superfamily member 11 | Extracellular Space | >80% | >80% |
| TNFSF12 (TEWAK) | TNF superfamily member 12 | Extracellular Space | >80% | >80% |
| TNFSF14 | TNF superfamily member 14 | Extracellular Space | >80% | >80% |
| TSLP | thymic stromal lymphopoietin | Extracellular Space | <20% | <20% |
| VEGFA | vascular endothelial growth factor A | Extracellular Space | >80% | >80% |

## **Supplementary Table S2. Detailed fold change and p-values of oligoJIA-healthy control cross-sectional analysis.**

| **Proteins** | **ΔNPX(Oligo-healthy)** | **-Log10(q-value)** |
| --- | --- | --- |
| **SIRT2** | **-1.255** | 3.69897 |
| **STAMBP** | **-1.082** | 2.65757732 |
| **CXCL5** | **-1.36** | 2.50863831 |
| **CXCL6** | **-1.043** | 2.26760624 |
| **SULT1A1** | **-1.191** | 2.06048075 |
| EIF4EBP1 | -0.6159 | 1.55284197 |
| AXIN1 | -0.767 | 1.36653154 |
| **MMP1** | **1.169** | 1.36653154 |
| CASP8 | -0.9336 | 1.36653154 |
| CCL13 | -0.6687 | 1.30364361 |
| CXCL1 | -0.7316 | 1.18045606 |
| CCL8 | -0.6586 | 1.16685289 |
| IL18 | -0.5912 | 0.99826629 |
| IL7 | -0.6374 | 0.99012437 |
| IL6 | 0.9351 | 0.99012437 |
| S100A12 | 0.7102 | 0.77365791 |
| TNFSF14 | -0.3965 | 0.56177419 |
| CD40 | -0.4845 | 0.48771594 |
| CCL4 | -0.4564 | 0.32679472 |
| CD244 | -0.4115 | 0.30233484 |
| TGFB1 | -0.3217 | 0.28912138 |
| CCL11 | -0.2514 | 0.28912138 |
| CCL3 | -0.4194 | 0.25703933 |
| ADA | -0.2939 | 0.25555054 |
| CXCL9 | 0.4035 | 0.25041827 |
| CXCL11 | -0.2132 | 0.20045269 |
| IL8 | -0.1021 | 0.11148354 |
| KITLG | -0.3672 | 0.11019425 |
| IL10RA | 0.258 | 0.11019425 |
| CD274 | -0.181 | 0.11019425 |
| IL10 | 0.3446 | 0.11019425 |
| CCL28 | -0.2363 | 0.11019425 |
| CCL20 | -0.1142 | 0.11019425 |
| OPG | -0.15168 | 0.10640467 |
| CST5 | 0.238381 | 0.10640467 |
| OSM | 0.136293 | 0.10640467 |
| IL18R1 | -0.15137 | 0.10640467 |
| CCL19 | 0.302055 | 0.0712458 |
| TNFSF12 | -0.14981 | 0.0712458 |
| MMP10 | -0.08639 | 0.07052998 |
| VEGFA | -0.04225 | 0.06722193 |
| CDCP1 | 0.197282 | 0.06454312 |
| TNFRSF9 | 0.126708 | 0.06454312 |
| TNF | 0.175 | 0.06454312 |
| CD6 | -0.22133 | 0.05685187 |
| IL17A | -0.17093 | 0.05369515 |
| TNFSF11 | -0.04863 | 0.05369515 |
| PLAU | -0.07847 | 0.04832256 |
| CCL2 | 0.031488 | 0.04832256 |
| TNFSF10 | -0.04411 | 0.04832256 |
| TGFA | 0.079446 | 0.04832256 |
| FGF23 | -0.12094 | 0.04832256 |
| LIFR | -0.08055 | 0.04832256 |
| FGF21 | 0.09792 | 0.04832256 |
| IL15RA | 0.017387 | 0.04832256 |
| IL10RB | -0.01415 | 0.04832256 |
| HGF | 0.070166 | 0.04832256 |
| IL12B | 0.190448 | 0.04832256 |
| CCL23 | 0.035562 | 0.04832256 |
| CD5 | -0.08388 | 0.04832256 |
| FLT3LG | -0.15233 | 0.04832256 |
| FGF19 | 0.042825 | 0.04832256 |
| CCL25 | 0.009311 | 0.04832256 |
| CX3CL1 | -0.06726 | 0.04832256 |
| NTF3 | 0.016137 | 0.04832256 |
| TNFB | -0.07885 | 0.04832256 |
| CSF1 | 0.105076 | 0.04832256 |
| DNER | -0.06012 | 0.04832256 |
| CD8A | 0.09665 | 0.04832256 |
| CXCL10 | 0.082902 | 0.04200982 |

## **Supplementary Table S3. Gene ontology (GO) analysis results based on the 19 significantly different SF proteins between early and persistent phase of oligoJIA**

| **GO-Term** | **Description** | **Involved proteins** | **Strength*** | **p-value after FDR correction**** |
| --- | --- | --- | --- | --- |
| GO:2000503 | Positive regulation of natural killer cell chemotaxis | CCL3, CCL4, CCL7 | 2.71 | 1.14e-05 |
| GO:0140131 | Positive regulation of lymphocyte chemotaxis | CCL3, CCL4, CCL7, LIF | 2.29 | 1.85e-06 |
| GO:0048012 | Hepatocyte growth factor receptor signaling pathway | HGF, LIF | 2.23 | 0.0072 |
| GO:0043922 | Negative regulation by host of viral transcription | CCL3, CCL4 | 2.17 | 0.0092 |
| GO:0030593 | Neutrophil chemotaxis | CCL3, CCL4, CCL7, CXCL8, CXCL9, CXCL1, CXCL6, CCL20, CXCL5, S100A12 | 2.14 | 4.69e-16 |
| GO:2000403 | Positive regulation of lymphocyte migration | CCL3, CCL4, CCL7, CCL20, TNFSF14 | 2.13 | 1.46e-07 |
| GO:1900424 | Regulation of defense response to bacterium | CXCL6, SIRT2 | 2.11 | 0.0114 |
| GO:0035729 | Cellular response to hepatocyte growth factor stimulus | HGF, SIRT2 | 2.11 | 0.0114 |
| GO:0097530 | Granulocyte migration | CCL3, CCL4, CCL7, CXCL8, CXCL9, CXCL1, CXCL6, CCL20, CXCL5, S100A12, IL17A | 2.09 | 5.15e-17 |
| GO:0002548 | Monocyte chemotaxis | CCL3, CCL4, CCL7, CCL20, S100A12 | 2.08 | 2.33e-07 |
| GO:0070098 | Chemokine-mediated signaling pathway | CCL3, CCL4, CCL7, CXCL8, CXCL9, CXCL1, CXCL6, CCL20, CXCL5 | 2.06 | 7.34e-14 |
| GO:0048245 | Eosinophil chemotaxis | CCL3, CCL7 | 2.04 | 0.0151 |
| GO:1901741 | Positive regulation of myoblast fusion | CXCL9, TNFSF14 | 2.01 | 0.0164 |
| GO:0045663 | Positive regulation of myoblast differentiation | CXCL9, TNFSF14 | 1.93 | 0.0217 |
| GO:0048247 | Lymphocyte chemotaxis | CCL3, CCL4, CCL7, CCL20 | 1.92 | 3.00e-05 |

* Strength is calculated as log10(observed / expected). This measure describes how large the enrichment effect is. It’s the ratio between i) the number of proteins in your network that are annotated with a term and ii) the number of proteins that we expect to be annotated with this term in a random network of the same size.

** p-value after FDR correction: this measure describes how significant the enrichment is. Shown are p-values corrected for multiple testing within each category using the Benjamini–Hochberg procedure.

## **Supplementary Table S4. Kyoto Encyclopedia of Genes and Genomes (KEGG) analysis results based on the 19 significantly different SF proteins between early and persistent phase of oligoJIA**

| **Pathways** | **Description** | **Involved proteins** | **Strength*** | **p-value after FDR correction**** |
| --- | --- | --- | --- | --- |
| hsa04061 | Viral protein interaction with cytokine and cytokine receptor | TNFSF10, CXCL8, CXCL9, CCL4, CCL20, CCL3, CXCL1, CXCL5, CCL7, CXCL6, IL18R1, LIF | 2.11 | 3.45e-21 |
| hsa05323 | Rheumatoid arthritis | CXCL8, CCL20, CCL3, CXCL1, IL17A, CXCL5, CXCL6 | 1.93 | 1.62e-10 |
| hsa04657 | IL-17 signaling pathway | CXCL8, CCL20, CXCL1, IL17A, CXCL5, CXCL6, CCL7 | 1.89 | 2.19e-10 |
| hsa04060 | Cytokine-cytokine receptor interaction | TNFSF10, OSM, IL17A,CXCL8, CXCL9, CCL4, CCL20, CCL3, CXCL1, CXCL5, CCL7, CXCL6, IL18R1, LIF | 1.74 | 4.46e-22 |
| hsa04668 | TNF signaling pathway | LIF, IL18R1, CXCL1, CCL20, CXCL5, CXCL6 | 1.74 | 6.00e-08 |
| hsa04062 | Chemokine signaling pathway | CXCL8, CXCL9, CCL4, CCL20, CCL3, CXCL1, CXCL5, CCL7, CXCL6 | 1.70 | 7.58e-12 |
| hsa05144 | Malaria | HGF, CXCL8 | 1.65 | 0.0329 |
| hsa05133 | Pertussis | CXCL8, CXCL5, CXCL6 | 1.62 | 0.0020 |
| hsa04620 | Toll-like receptor signaling pathway | CCL3, CCL4, CXCL8, CXCL9 | 1.61 | 0.00014 |
| hsa04064 | NF-kappa B signaling pathway | CCL4, CXCL1, CXCL8, TNFSF14 | 1.61 | 0.00014 |
| hsa05134 | Legionellosis | CXCL1, CXCL8 | 1.57 | 0.0386 |
| hsa05321 | Inflammatory bowel disease | IL17A, IL18R1 | 1.54 | 0.0421 |
| hsa05120 | Epithelial cell signaling in Helicobacter pylori infection | CXCL1, CXCL8 | 1.49 | 0.0483 |
| hsa05163 | Human cytomegalovirus infection | CCL3, CCL4, CXCL8 | 1.15 | 0.0367 |

* Strength is calculated as log10(observed / expected). This measure describes how large the enrichment effect is. It’s the ratio between i) the number of proteins in your network that are annotated with a term and ii) the number of proteins that we expect to be annotated with this term in a random network of the same size.

** p-value after FDR correction: this measure describes how significant the enrichment is. Shown are p-values corrected for multiple testing within each category using the Benjamini–Hochberg procedure.


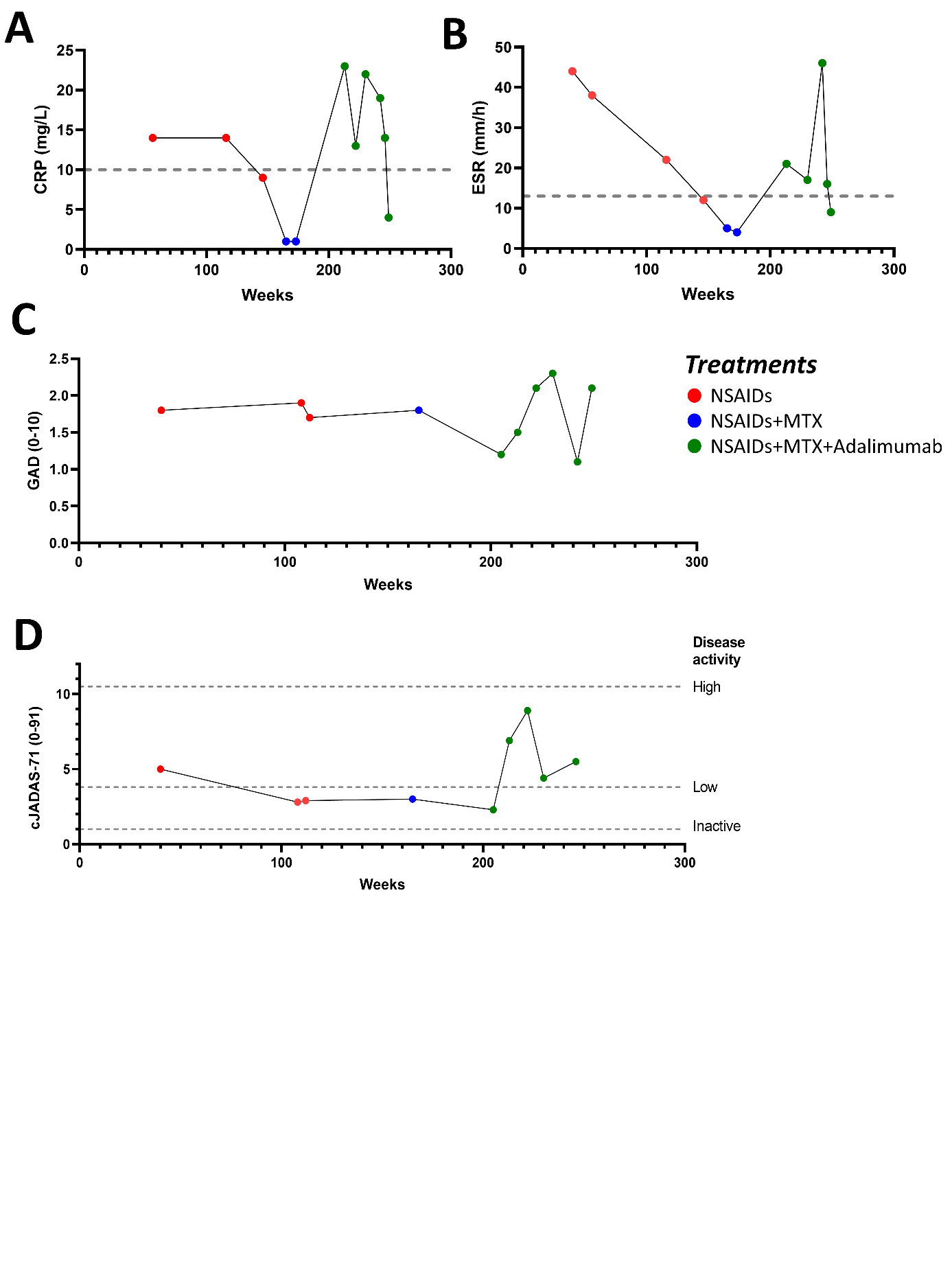


**Supplementary Fig. S1. Clinical parameters of the case study.** The clinical measurements, including C-reactive protein (CRP) (A), erythrocyte sedimentation rate (ESR) (B), Global Assessment scores by Doctor (GAD) (C) and Clinical Juvenile Arthritis Disease Activity Score of 71 joints (cJADAS-71) (D), were listed. Based on the age of the patient, the limitation and range of each parameter are: CRP higher than 10 mg/L or ESR higher than 13 mm/h is regarded as inflammation. GAD score ranges between 0 and 10. cJADAS-71 score ranges between 0 and 91, the high, low and inactive disease activity cut-off are 10.5, 3.8 and 1.0, separately.
